# 

**Published:** 1877-03

**Authors:** 


					﻿ESTABLISHED IN 1855.
Volume XIV j MARCH-1877 j Numbeu 12.	(
ATLANTA
Medical and Surgical Journa.
MONTHLY: THllKE LOLLARS PER ANNCM. IN ADVANCE.
EDITOR ;
J.	(}. WRSTMOKHl.AXn, M.D.
Pri/fesMor iltiferui Mnlit'a in Aflnnin Mfili/'al Ooilfii/f.
AH.SO<'IATE EDITOR :
K.	W. AVESTMOIIEI.AXI), M.I).
lienifin.'ih'fiior anti VroKfctor of AnnlMiii/ i)t Atbinla Mf'iliffil I'nileijp.
H. H. DICKSON, PUBLISHER.
/MX ET SdENTlA, SED VEIUTAS SINE T!AI<)HE.
A'J IrAN'l’A. OEOROIA:
}!. H. bK'KShV. HhbK iNh JOIl rH/XTKK Ay/> Hf»tKPJXDF.K.
1877.
				

